# Supplementary material for: The influence of sample distribution on growth model output for a highly-exploited marine fish, the Gulf Corvina (Cynoscion othonopterus)
Source: PeerJ. 2018 Sep 17;6:e5582. doi: 10.7717/peerj.5582 (PMC6148420; doi:10.7717/peerj.5582)
Supplement: Supplemental Information 1 [file peerj-06-5582-s001.docx]

**Supplementary information**

**Appendix S1. Details of the per-recruit model developed for Gulf Corvina (*Cynoscion othonopterus*).**

We developed a parsimonious, non-spatial, per-recruit model for Gulf Corvina (*Cynoscion othonopterus*), which estimates the female spawning-stock-biomass-per-recruit (SSBR) and yield-per-recruit (YPR) of the species in relation to the annual exploitation rates of its old adults (≥ 5 year-old individuals; $E_{OA}$). Our model assumes that Gulf Corvina is harvested only during the spawning season (based on the results of Erisman et al. (2012b)), and that all the juveniles and young adults of Gulf Corvina (i.e., 0-4 years old individuals) escape fishing and the annual fishing mortality rate of these life stages due to bycatch is negligible (based on Walsh et al. (2004), Erisman et al. (2012a) and Pérez-Valencia (2012)). Our model also makes the assumption that the “encierre” technique used by the Gulf Corvina fishery means that there is no upper size limit to the fishery (i.e., large individuals do not escape capture); this assumption is supported by several lines of evidence, as described in detail in Erisman et al. (2014).

In the following, we first present the alternative assumptions we made regarding the growth in length and weight of Gulf Corvina in the per-recruit model. Then, we detail the calculation of SSBR, YPR and the current value of $E_{OA}$ for Gulf Corvina. Finally, we describe the estimation of reference points for the species.

**Growth in length and weight**

In the per-recruit model, Gulf Corvina is assumed to grow according to one of five alternative growth models (Figure S1): (1) the von Bertalanffy model developed in Gherard et al. (2013), referred to as the “Gherard model”; (2) the von Bertalanffy model fit to raw data in the present study; (3) the von Bertalanffy model fit to raw data bolstered by simulation values in this study; (4) the Schnute-Richards model fit to raw data in the present study; and (5) the Schnute-Richards model fit to raw data bolstered by simulation values in this study. The Gherard model assumes the following relationship between length $L$ (in mm TL) and age $a$ (in years):

Eq. S1.1: $L\left( a \right)=1006*[1-e^{-0.225\left( a-0.616 \right)}]$

The body weight of Gulf Corvina at age *a*, $w\left( a \right)$, is calculated from the length-at-age predicted by one of the five growth models as follows (Figure S2; Gherard et al., 2013):

Eq. S1.2: $w\left( a \right)=2*{10}^{-5}*{L\left( a \right)}^{2.8834}$

**Estimation of SSBR, YPR and the current value of** $\boldsymbol{E}_{\boldsymbol{OA}}$

Our per-recruit model estimates SSBR as follows:

Eq. S1.3: $SSBR=\frac{SR}{1+SR}\int_{a_{R}}^{a_{OA}} e^{-M\left( a_{R}-0 \right)}e^{-M\left( {a-a}_{R} \right)}w\left( a \right)da$

$$+\frac{SR}{1+SR}\int_{a_{OA}}^{a_{MAX}} e^{-M\left( a_{R}-0 \right)}e^{-M\left( {a_{OA}-a}_{R} \right)}\left[ e^{-M\left( {a-a}_{OA} \right)}\left( 1-E_{OA} \right)^{\left( {a-a}_{OA} \right)} \right]w\left( a \right)da$$

where $SR$ is the female:male sex ratio of Gulf Corvina (which is assumed to be 1:1 for the sake of simplicity); $M$ is the natural mortality rate (0.28 year^-1^; estimated in Erisman et al. (2014) using Pauly (1980)’s relationship); $E_{OA}$ is the exploitation rate of old adults (in year^-1^) (and, therefore, $\left( 1-E_{OA} \right)$ is the escapement rate of old adults (in year^-1^)); $a_{R}$ is the age of sexual maturity (2 years; Gherard et al., 2013); $a_{OA}$ is the age of transition from the young adult stage to the old adult stage (i.e., 5 years); and $a_{MAX}$ is the maximum age (9 years; Gherard et al., 2013). It is reasonable to use exploitation rates rather than the more conventional fishing mortality rates for young and old adults of corvina, because the Gulf Corvina fishing season is very short (Erisman et al., 2014).

YPR is estimated by our pre-recruit model as follows:

Eq. S1.4: $YPR=$

$$E_{OA}\int_{a_{OA}}^{a_{MAX}} e^{-M\left( a_{R}-0 \right)}e^{-M\left( {a_{OA}-a}_{R} \right)}\left[ e^{-M\left( {a-a}_{OA} \right)}\left( 1-E_{OA} \right)^{\left( {a-a}_{OA} \right)} \right]w\left( a \right)da$$

To determine the current value of $E_{OA}$ for Gulf Corvina, we compiled length data from 2013-2015 to create a length frequency distribution. Then, we determined that $E_{OA}$ is equal to 0.825 year^-1^, based on combinations of $M$ (Pauly, Hoenig) and three methods to calculate the total mortality rate of Gulf Corvina (Seine, LDFA, catch curves). Calculation of this rate is explained in greater detail in Erisman et al. (2014).

**Estimation of reference points**

So as to facilitate the discussions of our results, we estimated two reference points for Gulf Corvina with our per-recruit model: the maximum value of the YPR of Gulf Corvina, YPR_max_; and the natural SSBR of Gulf Corvina (NSSBR), i.e., its SSBR in the absence of fishing (Figures S3 and S4).

**References of Appendix S1**

Erisman, B., Aburto-Oropeza, O., Apel, A., Fujita, R., 2012a. An assessment of risks to the corvina golvina fishery. Report prepared for the Corvina Technical Working Group. 18 pp.

Erisman, B., Aburto-Oropeza, O., Gonzalez-Abraham, C., Mascareñas-Osorio, I., Moreno-Báez, M., Hastings, P.A., 2012b. Spatio-temporal dynamics of a fish spawning aggregation and its fishery in the Gulf of California. Scientific Reports 2, 284.

Erisman, BE., Apel, AM., MacCall, AD., Román, MJ., Fujita, R., 2014. The influence of gear selectivity and spawning behavior on a data-poor assessment of a spawning aggregation fishery. Fisheries research 159, 75–87.

Gherard, KE., Erisman, BE., Aburto-Oropeza, O., Rowell, K., Allen, LG., 2013. Growth, development, and reproduction in Gulf corvina (*Cynoscion othonopterus*). Bulletin, Southern California Academy of Sciences 112, 1–18.

Pauly D., 1980. On the interrelationships between natural mortality, growth parameters, and mean environmental temperature in 175 fish stocks. ICES Journal of Marine Science 39, 175–192.

Pérez-Valencia, S. A. 2012. Manifestación de Impacto Ambiental para la pescariberena responsable en la Reserva de la Biosfera Alto Golfo de California y Deltadel Río Colorado: Costa Este. El Golfo de Santa Clara: Centro Intercultural deEstudios de Desiertos y Océanos, A.C. 264 pp.

Walsh, P., Grant, S., Winger, P., Blackwood, G., Balmori-Ramirez, A., Silva-Ramírez, T., 2004. An investigation of alternative harvesting methods to reduce the by-catch of Vaquita porpoise in the Upper Gulf of California shrimp gillnet fishery. Unpublished report prepared for World Wildlife Fund–US, Washington, DC.

**Appendix S2. 95% confidence intervals for the growth parameters estimated from raw data.**

The 95% confidence intervals for the von Bertalanffy, Gompertz, logistic, Schnute and Schnute-Richards growth parameters estimated from raw data are provided in Table S1, S2, S3, S4 and S4, respectively.
