# Supplementary figures and images for: The influence of sample distribution on growth model output for a highly-exploited marine fish, the Gulf Corvina (Cynoscion othonopterus)
Source: PeerJ. 2018 Sep 17;6:e5582. doi: 10.7717/peerj.5582 (PMC6148420; doi:10.7717/peerj.5582)

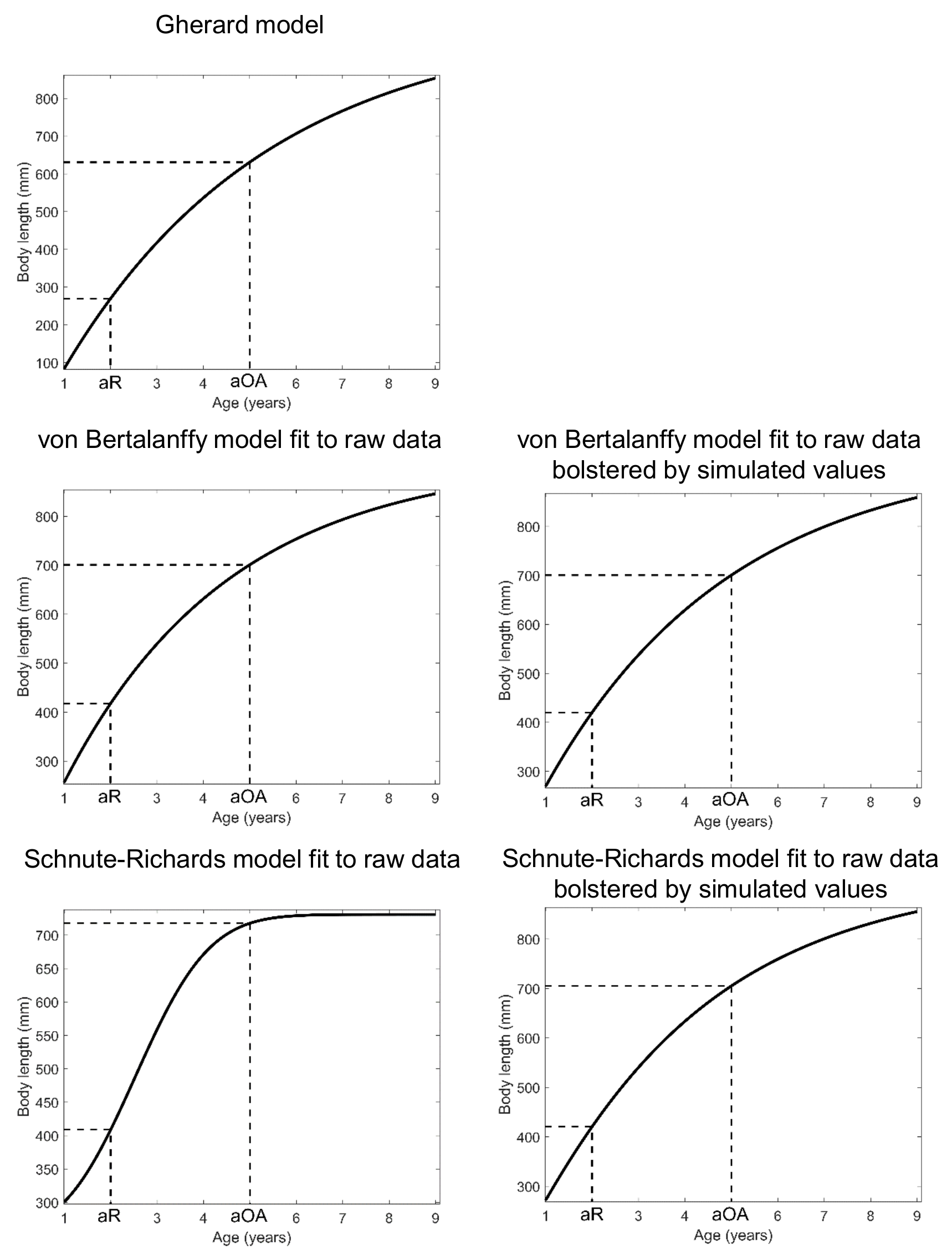

Supplement: Supplemental Information 2 — aR = age of sexual maturity (2 years; Gherard et al., 2013) - aOA = age of transition from the young adult stage to the old adult stage (5 years). [file peerj-06-5582-s002.png]

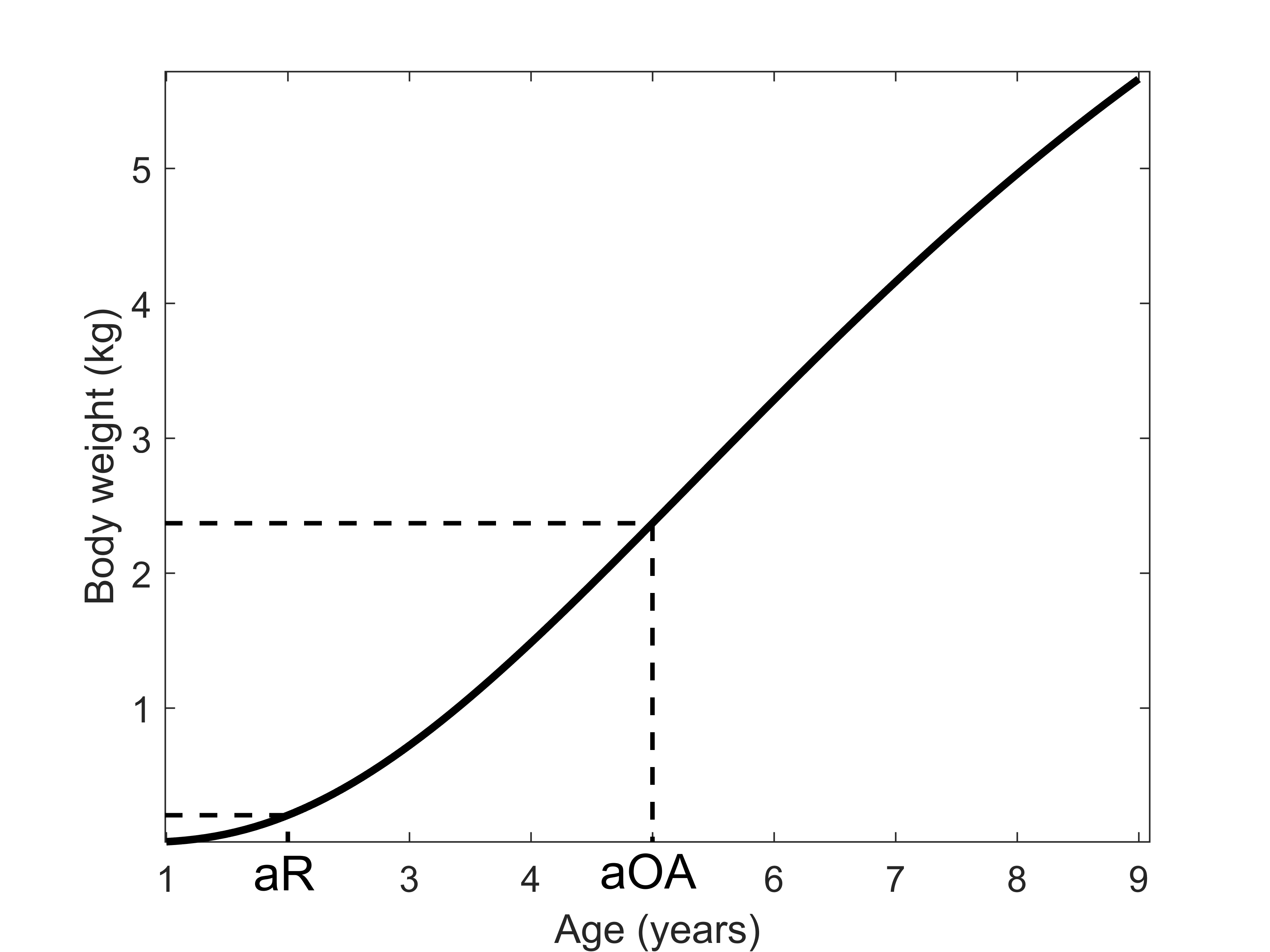

Supplement: Supplemental Information 3 — aR = age of sexual maturity (2 years; Gherard et al., 2013) - aOA = age of transition from the young adult stage to the old adult stage (5 years). [file peerj-06-5582-s003.png]

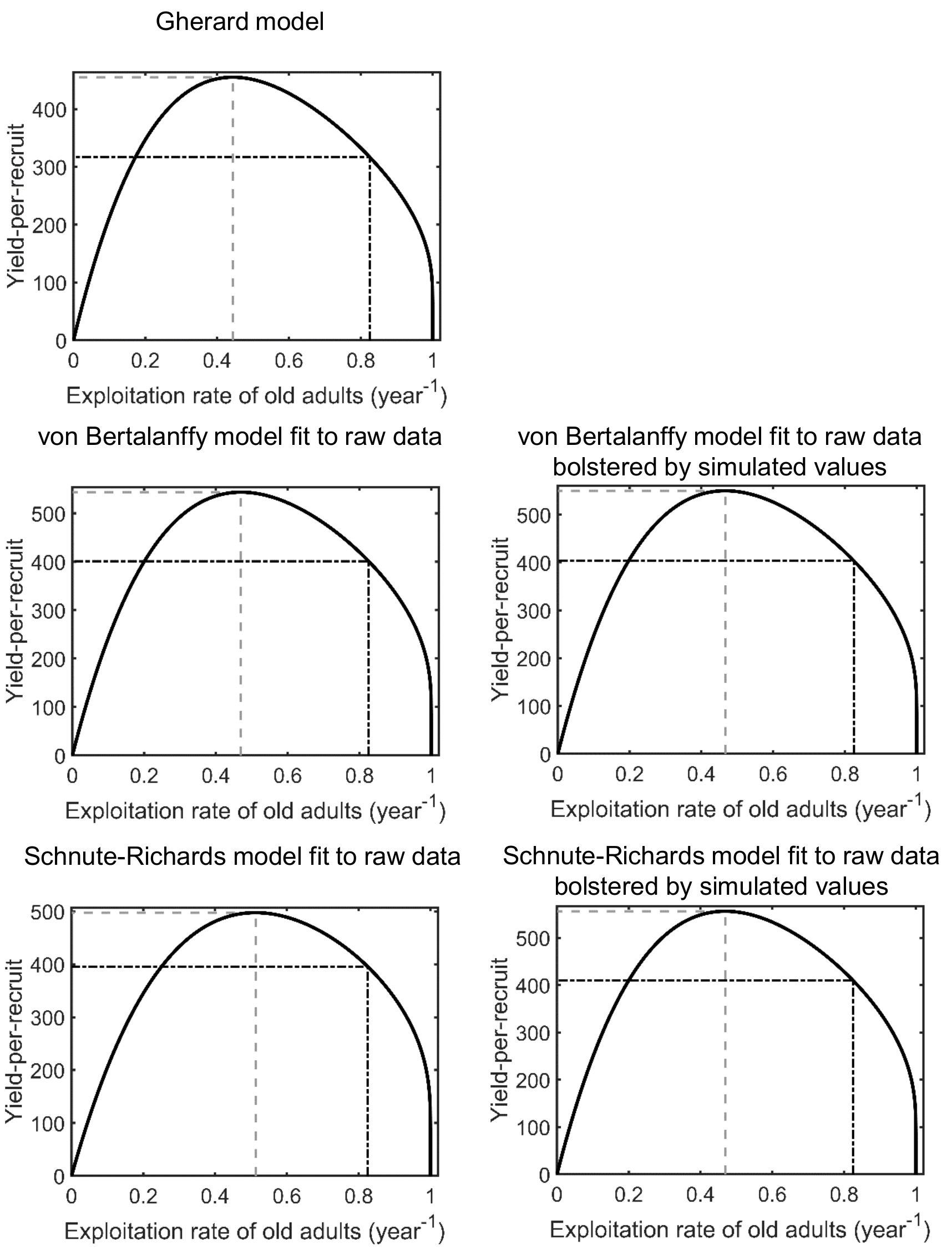

Supplement: Supplemental Information 4 — In each panel, the dashed-dotted black lines indicate the current exploitation rate of old adults of Gulf Corvina and the corresponding value of yield-per-recruit. Moreover, in each panel, the dashed grey lines indicate the exploitation rate of old adults of Gulf Corvina at which the yield-per-recruit of the species reaches a maximum and the corresponding value of yield-per-recruit. [file peerj-06-5582-s004.png]

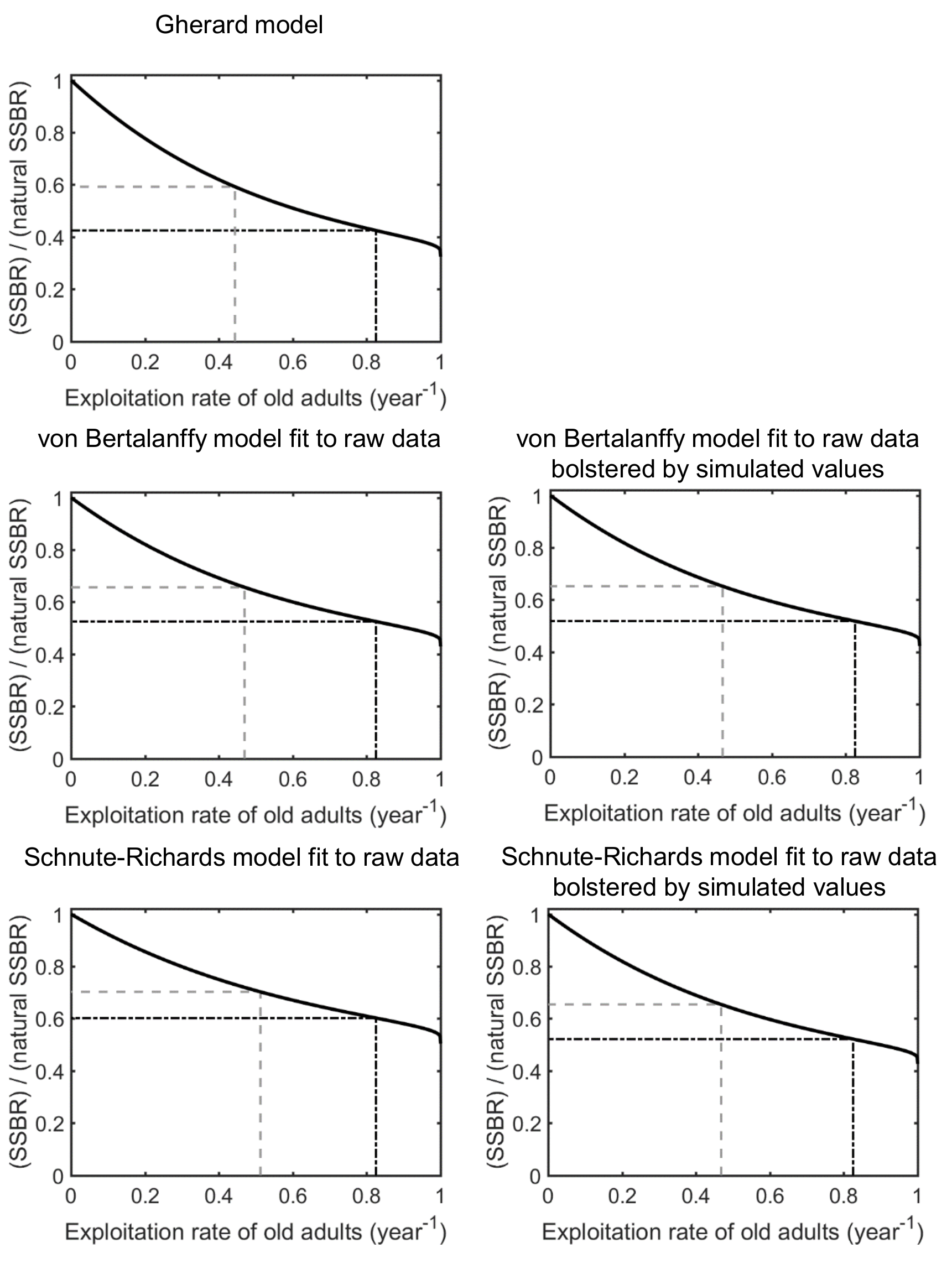

Supplement: Supplemental Information 5 — In each panel, the dashed-dotted black lines indicate the current exploitation rate of old adults of Gulf Corvina and the corresponding value of yield-per-recruit. Moreover, in each panel, the dashed grey lines indicate the exploitation rate of old adults of Gulf Corvina at which the yield-per-recruit of the species reaches a maximum and the corresponding value of yield-per-recruit. [file peerj-06-5582-s005.png]
