# Supplementary material for: The influence of sample distribution on growth model output for a highly-exploited marine fish, the Gulf Corvina (Cynoscion othonopterus)
Source: PeerJ. 2018 Sep 17;6:e5582. doi: 10.7717/peerj.5582 (PMC6148420; doi:10.7717/peerj.5582)
Supplement: Table S1 [file peerj-06-5582-s006.docx]

| Parameter | Estimate | 95% lower CI | 95% upper CI |
| --- | --- | --- | --- |
|  |  |  |  |
| *L_∞_* | 916.048 | 858.953 | 989.574 |
| *K* | 0.281 | 0.233 | 0.333 |
| *t_0_* | -0.170 | -0.365 | -0.009 |
|  |  |  |  |
