# Supplementary material for: The influence of sample distribution on growth model output for a highly-exploited marine fish, the Gulf Corvina (Cynoscion othonopterus)
Source: PeerJ. 2018 Sep 17;6:e5582. doi: 10.7717/peerj.5582 (PMC6148420; doi:10.7717/peerj.5582)
Supplement: Table S2 [file peerj-06-5582-s007.docx]

| Parameter | Estimate | 95% lower CI | 95% upper CI |
| --- | --- | --- | --- |
|  |  |  |  |
| *L_∞_* | 820.639 | 793.079 | 859.285 |
| *K* | 0.512 | 0.451 | 0.571 |
| *t_0_* | 1.293 | 1.206 | 1.402 |
|  |  |  |  |
