# Supplementary material for: The influence of sample distribution on growth model output for a highly-exploited marine fish, the Gulf Corvina (Cynoscion othonopterus)
Source: PeerJ. 2018 Sep 17;6:e5582. doi: 10.7717/peerj.5582 (PMC6148420; doi:10.7717/peerj.5582)
Supplement: Table S4 [file peerj-06-5582-s009.docx]

| Parameter | Estimate | 95% lower CI | 95% upper CI |
| --- | --- | --- | --- |
|  |  |  |  |
| *a* | 3.356 | 2.761 | 4.277 |
| *b* | -0.325 | -0.479 | -0.216 |
|  |  |  |  |
