# Supplementary material for: The influence of sample distribution on growth model output for a highly-exploited marine fish, the Gulf Corvina (Cynoscion othonopterus)
Source: PeerJ. 2018 Sep 17;6:e5582. doi: 10.7717/peerj.5582 (PMC6148420; doi:10.7717/peerj.5582)
Supplement: Table S5 [file peerj-06-5582-s010.docx]

| Parameter | Estimate | 95% lower CI | 95% upper CI |
| --- | --- | --- | --- |
|  |  |  |  |
| *L_∞_* | 730.910 | 715.188 | 748.179 |
| *α* | -0.003 | -0.750 | 8.466 |
| *a* | 0.117 | 0.041 | 0.394 |
| *b* | 0.003 | -2.054 | 1.287 |
| *c* | 2.181 | 1.612 | 2.814 |
|  |  |  |  |
